# Child Distress Expression and Regulation Behaviors: A Systematic Review and Meta-Analysis

**DOI:** 10.3390/children9020174

**Published:** 2022-02-01

**Authors:** Hannah G. Gennis, Oana Bucsea, Shaylea D. Badovinac, Stefano Costa, C. Meghan McMurtry, David B. Flora, Rebecca Pillai Riddell

**Affiliations:** 1 Department of Psychology, York University, 4700 Keele Street, Toronto, ON M3J 1P3, Canada; hgennis@yorku.ca (H.G.G.); obucsea@yorku.ca (O.B.); sdbadov@yorku.ca (S.D.B.); stefano@voldock.com (S.C.); dflora@yorku.ca (D.B.F.); 2 Department of Psychology, University of Guelph, 50 Stone Road East, Guelph, ON N1G 2W1, Canada; cmcmurtr@uoguelph.ca; 3 Pediatric Chronic Pain Program, McMaster Children’s Hospital, 1200 Main Street West, Hamilton, ON L8N 3Z5, Canada; 4 Children’s Health Research Institute, 345 Westminster Avenue, London, ON N6C 4V3, Canada; 5 Department of Pediatrics, Western University, 800 Commissioners Road East, London, ON N6A 5W9, Canada; 6 Department of Psychiatry, University of Toronto, 250 College Street, Toronto, ON M5T 1R8, Canada; 7 Department of Psychiatry Research, The Hospital for Sick Children, 555 University Avenue, Toronto, ON M5T IR8, Canada

**Keywords:** distress, emotion regulation, behavior, infant, toddler

## Abstract

The goal of the current study was to review and meta-analyze the literature on relationships between child distress expression behaviors (e.g., cry) and three clusters of child distress regulation behaviors (disengagement of attention, parent-focused behaviors, and self-soothing) in the first three years of life. This review was registered with PROSPERO (CRD42020157505). Unique abstracts were identified through Medline, Embase, and PsycINFO (*n* = 13,239), and 295 studies were selected for full-text review. Studies were included if they provided data from infants or toddlers in a distress task, had distinct behavioral measures of distress expression and one of the three distress regulation clusters, and assessed the concurrent association between them. Thirty-one studies were included in the meta-analysis and rated on quality. Nine separate meta-analyses were conducted, stratified by child age (first, second, and third year) and regulation behavior clusters (disengagement of attention, parent-focused, and self-soothing). The weighted mean correlations for disengagement of attention behaviors were −0.28 (year 1), −0.44 (year 2), and −0.30 (year 3). For parent-focused behaviors, the weighted mean effects were 0.00 (year 1), 0.20 (year 2), and 0.11 (year 3). Finally, the weighted mean effects for self-soothing behaviors were −0.23 (year 1), 0.25 (year 2), and −0.10 (year 3). The second year of life showed the strongest relationships, although heterogeneity of effects was substantial across the analyses. Limitations include only analyzing concurrent relationships and lack of naturalistic distress paradigms in the literature.

## 1. Introduction

The term emotion regulation has been widely used in various social and emotional contexts, as well as across different developmental periods [1]. Emotion regulation reflects one’s ability to monitor, evaluate, and modify emotions to attain a goal [2]. Inherent in this definition is that emotions can be regulated, or modified, via different social, behavioral, cognitive, or biological processes [1,2,3]. The focus of this review will be on behaviors.

There is evidence of self-regulation as early as the first few days of life. Prior to three months, behaviors are largely categorized by unintentional, non-planned motor actions. Neonates lack the cognitive and motor abilities needed to move away from a distressing stimulus, and therefore are speculated to shut out stimulation by closing their eyes, turning their head reflexively, using thumb to mouth actions, sucking, and crying [4,5]. Crying is understood as a reflexive response to distress that draws the caregiver close [6]. In these early months, distress regulation is often required in the context of physiological distress (e.g., hunger or pain). During painful experiences (e.g., vaccination), for example, research has shown that younger infants shut their eyes following a painful stimulus (e.g., a needle), and as infants age, they tend to open their eyes earlier [7]. Sroufe [8] has discussed that by three months, infants show their first “true emotional reactions”.

Caregivers are particularly important for distress regulation in the first year, because while infants are developing the ability to enact certain regulatory behaviors, their skill set is inadequate to meet all the novel stressors they encounter or alleviate the stressor itself [5]. An infant’s ability to regulate from distress is heavily embedded in co-regulation with the caregiver, and that co-regulation forms the underlying canvas on which self-regulation is learned [4,5,8]. Sameroff [9] describes these developmental processes in terms of a shift from a “other-regulation” (i.e., regulation provided by caregivers) to “self-regulation” that occurs as infants take on an increasing role in managing their own distress beginning in toddlerhood.

Rothbart et al. [10] have outlined developmental trends in self-regulatory behaviors based on observing young infants into their toddler years under several different distressing and non-distressing conditions. Their work has shown that as infants age, they tend to move from more physical regulatory behaviors (e.g., mouthing and sucking) to more active forms of regulatory behaviors. Between 3 and 4 months, there is a hypothesized shift toward disengagement of attention that is solidified by 6 months of age [10]. As the first year progresses, children are considered “true emotional beings” [11], and regulatory efforts become more purposeful. Infants’ use of emotion regulation behaviors increases dramatically by the end of the first year due to changes in motor and visual systems, as well as social and emotional domains [4,5].

As children enter toddlerhood (i.e., the second year), emotion regulation strategies are largely social and include signalling to the caregiver for support in regulation [8]. This is consistent with attachment theory [6], which suggests that when infants become distressed, they are innately driven to seek proximity with their caregivers for distress reduction. This process tends to solidify by 12 months of age [6,12,13]. In line with these findings, Rothbart et al. [10] has shown that as children age into their second year (13.5 months in this particular sample), toddlers’ attention becomes increasingly focused on their mothers. Further, continued changes in cognitive and motor processes allow toddlers to shift from more simple methods of attentional control (e.g., gaze aversion) [14,15] to more effortful redirection [16,17]. For example, one study examining the differences in frequency of object orientation during a frustration task found an increase in use of object orientation from four months to 16 months [18].

By the end of the second year, there is evidence of less reliance on the caregiver and more independent methods of emotion regulation, as toddlers begin to develop an understanding of causes of distress and how to use actions to alter or remove the cause [4,5]. It is important to note that while toddlers are able to self-regulate without caregiver intervention, there is still reliance on caregivers, who can serve as a supportive presence that enables the child’s self-regulation when demands may be too high [8]. In the third year, toddlers acquire an ability known as mentalization [19]. They begin to understand that their experience of emotion is different from those around them, and with the acquisition of language, begin to talk about their own and other people’s feelings [19]. This has important implications for emotion regulation behaviors.

The study of emotions and emotion regulation has been fruitful yet challenged by a difficulty in distinguishing emotional expression from emotion regulation. Independent assessment of emotion expression and regulatory behaviors was deemed to be a key methodological direction needed to advance the study of emotion regulation [1]. With this concept in mind and Thompson’s [2] definition of emotion regulation, it is not enough to consider a reduction in distress (i.e., emotional expression) as evidence of emotion regulation. Without looking at the relationship of particular emotion regulation behaviors on the emotional expression, true regulation is not evident. This review provides an analysis of the literature to date examining the relationship between distress expression and distress regulation behaviors in young children, in order to gain a firm understanding of how particular behaviors impact distress expression across the literature.

Based on our current review of the literature, there appears to be evidence of two distinct types of emotion regulation behaviors in infancy and toddlerhood: caregiver-directed self-regulation behaviors and independent self-regulation behaviors. Caregiver-directed behaviors, defined here as behaviors that solicit parent support to regulate distress, would include behaviors such as orienting to the parent and seeking proximity to the parent. Independent self-regulation behaviors would therefore be those that are not directed at the caregiver or “other”. These may include self-directed physical self-soothing (e.g., thumb sucking, auto-manipulative behaviors) or disengagement of attention (e.g., gaze aversion, focusing on a different object). Further, within the category of independent self-regulation behaviors, there appears to be a developmental shift from the use of more physical self-soothing strategies in infancy, to more attention-related strategies in toddlerhood. Thus, these were separated to gain a fuller understanding of these strategies.

The primary goal of this paper was to provide a synthesis of the concurrent relationships between distress expression and three unique clusters of emotion regulation strategies in the first three years of life. These three clusters are: 1. disengagement of attention behaviors, referring to any shift in attention that does not involve the parent (e.g., playing with a toy or object and averting gaze); 2. parent-focused behaviors, referring to any behavior a child does to get the parent’s attention or bring the parent close (e.g., gazing at the parent and vocalizations directed at the parent); 3. self-soothing behaviors, referring to the infant’s physical attempts to calm down (e.g., thumb sucking). The focus on assessing the relationships between distinct measures of emotion expression and distinct measures of emotion regulation strategies was based on Cole et al.’s statement of the importance of separating emotional valence and emotion regulation strategies as unique constructs [1].

There is a large body of literature providing data on the relationship between distress expression and distress regulation behaviors; however, there has yet to be a meta-analysis of these findings. Meta-analyses provide an objective, quantitative method to summarize a large literature on a given effect (e.g., mean associations between regulation behaviors and distress expression), while also including methods to understand factors contributing to the heterogeneity of effects across the literature [20]. Another benefit of meta-analyses is that they provide an accessible summary of a particular literature. Given the importance of emotion expression and emotion regulation to a number of scientific areas interested in child development (e.g., health psychology), this is a helpful starting point for researchers in broader research areas.

It is important to state that Cole et al. [1] emphasized additional methodological considerations not directly addressed in this review. These include notably the need to look at temporal or dynamic relationships between these two constructs. While the current review only focused on concurrent relationships to provide a general synthesis of the relationships between emotion expression and regulation behaviors, it does not negate the importance of taking a dynamic, time-based approach. Given the challenges with synthesizing over several time-based or predictive analyses, inclusion of such studies went beyond the scope of this particular review. Assessing the concurrent relationships is seen as an important first step in this synthesis, with temporal relationships needing to be addressed in future studies.

Further, an a priori decision was made to not extend the analyses into the preschool phase of early childhood (i.e., 4–5 years old). This was done for two reasons. First, there is a substantial shift from guided self-regulation (i.e., caregiver support present) in infancy and toddlerhood to less guided self-regulation in preschool. Second, the types of tasks and expectations (i.e., self-control and self-organization) of preschool [8] are qualitatively different in their demands.

## 2. Materials and Methods

### 2.1. Protocol and Registration

This review followed an a priori protocol according to the Preferred Reporting Items for Systematic Reviews and Meta-Analyses (PRISMA) guidelines [21]. The review protocol was pre-registered on the International Prospective Register of Systematic Reviews (PROSPERO) [22] prior to data extraction (CRD42020157505; https://www.crd.york.ac.uk/PROSPERO/display_record.php?RecordID=157505; accessed on 6 November 2019). After examining the quantity of data available, two deviations from the PROSPERO protocol occurred: (1) this study did not analyze the predictive relationships between distress expression and distress regulation behaviors (only concurrent); (2) this study did not analyze relationships that had a physiological distress outcome. See Supplementary File S1 for PRISMA Checkist.

### 2.2. Eligibility Criteria

Included studies were required to: (1) be English-language observational human cohort or cross-sectional studies; (2) have participants between the ages of 3 months 0 days and 35 months 31 days; (3) include a distressing task; (4) have separate behavioral measures of both distress expression (e.g., facial expressions and crying) and distress regulation behaviors (disengagement of attention, parent-focused, or self-soothing); and (5) report concurrent relationships between the two measures (i.e., measured within the same epochs). Prospective and longitudinal analyses were not considered for this review, due to the heterogeneity in analyses which complicated obtaining comparable effect sizes. Review articles, dissertations, case studies, commentaries, and conference abstracts were also excluded to focus on published peer-reviewed empirical work.

### 2.3. Systematic Search

A systematic search was conducted using Medline, Embase, and PsycINFO in December 2020 for English-language references published in the last 30 years. Search terms related to distress, emotion regulation, and infancy or toddlerhood were systematically paired (see Supplementary File S2 for search strategies).

### 2.4. Study Selection

Four independent coders rated 20% of total abstracts (based on the initial search results of 11,996 abstracts). Percentage agreement of abstract inclusion across pairs of coders ranged from 96.24% to 97.64%. Checks for percentage agreement were continued on subsets of abstracts throughout the remaining screening. Covidence software (www.covidence.org; accessed on 30 December 2019) was used for independent abstract rating.

### 2.5. Data Collection Process

Once full texts were included, all data were extracted by two reviewers using standardized forms. Study authors were contacted if data were missing. Discrepancies were minimal and resolved through discussion.

#### 2.5.1. Data Items

For each article, we recorded the country where the study took place, sample size, participant age in years (First year of life: 3 months 0 days to 11 months 30/31 days; Second year of life: 12 months 0 days to 23 months 30/31 days; and Third year of life: 24 months 0 days to 35 months 30/31 days), distress task, measure of distress expression, measure of distress regulation behaviors, and the correlation between the distress expression measure and the distress regulation measure. Studies were coded as including a fear task, a frustration task, and/or a task that invoked “other distress” (e.g., a competing demands task or exposure to another child’s or experimenter’s distress). If not explicitly specified as fear or frustration in the article, we coded any task including separation, flattening of affect, or ignoring as fear and coded any task involving a barrier or restraint as frustration. As shown in Table 1, two studies exposed the child to another individual’s distress—either the cry sound of a peer [23], or experimenter distress [24]. These two studies were categorized under fear. One study had a competing demands task [25], which was categorized under frustration. Further, if a study provided several measures of distress regulation behaviors in a particular category, composite measures were prioritized, followed by the behavior that most closely matched other behaviors that would be included in the same analytical category.

#### 2.5.2. Handling of Multiple Effect Sizes

Several studies included multiple effect sizes for one or more of the following reasons: (1) participants of a particular age group underwent two separate distress tasks; (2) there were two or more distress variables; (3) there were separate data from participating in a task with both mother and father; and (4) children participated at multiple ages. Further, several articles presented on the same sample. In these cases, effect sizes were selected from the most distressing task (based on distress scores or reviewing authors consensus), the distress task with the mother, or the effect size that most closely resembled the methodologies of the other included studies to minimize heterogeneity among studies. For example, when effect sizes from multiple ages were provided, we selected the age that was most similar to other studies in the meta-analysis to promote conceptual consistency among the effects meta-analyzed.

### 2.6. Risk of Bias/Quality Assessment

To evaluate the quality of evidence in the meta-analysis, a modification of the checklists designed by the National Heart, Blood, and Lungs Institute [26], Downs and Black [27], and Crombie [28] was used. The National Heart, Blood, and Lungs Institute has provided a guideline for assessing the quality of observational cohort and cross-sectional studies [26], and the Downs and Black [27] and Crombie [28] measures were chosen based on a multidisciplinary collaborative review discussing quality in case–control, cohort, and cross-sectional studies [29]. Quality items were scored as Yes, No, or Not applicable. Articles were consensus-coded for quality scores to ensure reliability. Disagreements between two raters were minimal (reliability was 94.8%) and resolved through discussion. There was a total of 16 items on the quality checklist, and a proportion score was calculated for each study based on the number of items endorsed (i.e., scored as Yes) out of the total applicable items (i.e., scored as either Yes or No). See Supplementary File S3 for Quality Measure.

### 2.7. Data Synthesis

The extracted data were stratified by age (first, second, and third year of life) and cluster of emotion regulation behavior (parent-focused strategies, self-soothing strategies, and disengagement of attention strategies), with a separate meta-analysis conducted for each of the resulting nine datasets.

#### Meta-Analysis

Correlations between distress expression and distress regulation behaviors were the summary effect size statistic used in the current meta-analysis. In one study [30], the distress expression measure reported was latency to distress (i.e., the amount of time until distress is displayed), for which the direction of the correlation was reversed.

Once correlations were obtained, Fisher’s *r*-to-*Z* transformation [31] was used to account for the non-normal sampling distribution of *r*. These *Z* statistics were then meta-analyzed, and the pooled weighted *Z* statistics were returned to *r* values for interpretation. Random-effects models were estimated because it is assumed that the true effect can vary from study to study (i.e., studies can differ on other factors beyond the random sampling of participants). The meta-analyses were conducted using the metafor package [32] in R, version 4.0.2 [33].

Cochran’s Q and I^2^ were used to describe heterogeneity among effect sizes (see [34]).

## 3. Results

### 3.1. Study Selection

**Figure 1:**
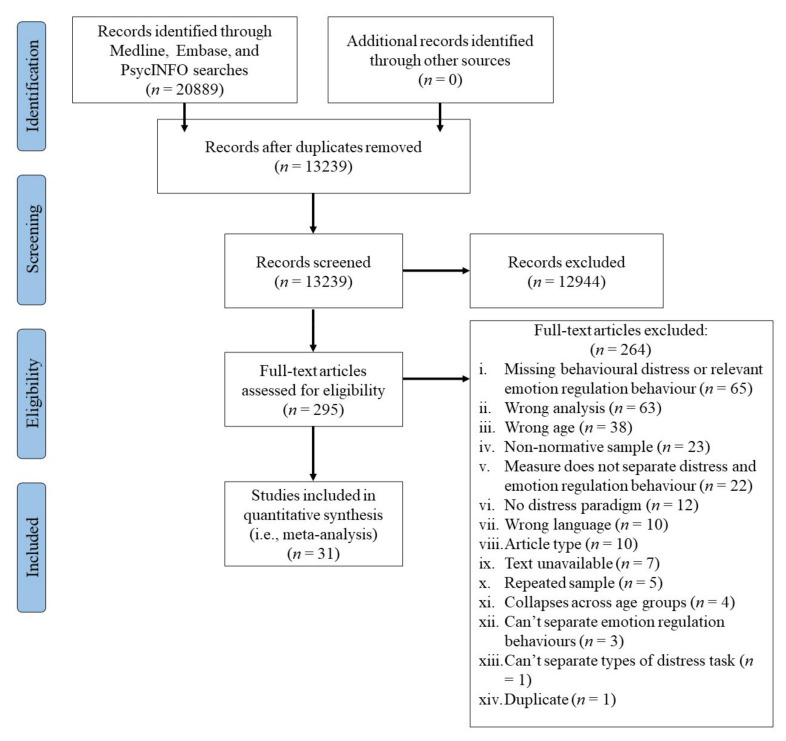
PRISMA flow diagram.

Over three search periods, 13,239 unique abstracts were identified, 295 articles were full-text reviewed, and 31 were included in the final meta-analyses. See Figure 1 for details on exclusion and reasons for exclusion for each stage.

### 3.2. Study Characteristics

Table 1 shows study characteristics including country of origin, sample size, distress task (specific task used), classification of distress task (fear or frustration), classification of distress regulation behavior, and study quality.

Most studies were from the United States (77%), with the rest coming from Canada, Italy, the Netherlands, Sweden, and Romania. Seven of the studies that assessed an age group in the first year of life provided data for disengagement of attention (43% frustration tasks, 57% fear tasks), 11 provided data for parent-focused strategies (36% frustration tasks, 64% fear tasks), and four provided data for self-soothing strategies (25% frustration tasks, 75% fear tasks). For the second year of life, 11 studies provided data for disengagement of attention (82% frustration tasks, 18% fear tasks), seven provided data for parent-focused strategies (71% frustration tasks, 29% fear tasks), and four provided data for self-soothing strategies (75% frustration tasks, 25% fear tasks). For the third year of life, five studies provided data for disengagement of attention (60% frustration tasks, 40% fear tasks), four for parent-focused strategies (75% frustration tasks, 25% fear tasks), and four for self-soothing strategies (25% frustration tasks, 75% fear tasks).

### 3.3. Quality Assessment

**Figure 2:**
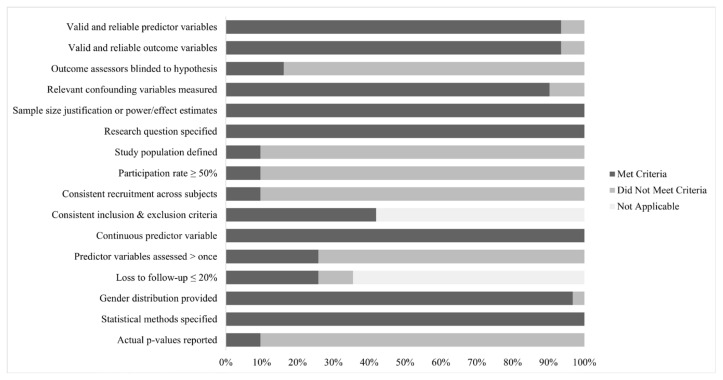
Quality assessment. Note. Bars represent the percentage of studies (out of 31) that fulfilled each criterion of the quality assessment.

Across the 31 studies, quality ratings ranged from 43% to 88%, with a mean rating of 62% and a median rating of 60%. Figure 2 outlines the percentage of studies that received credit (i.e., were coded as “Yes”) for each of the 16 items in the quality assessment.

Item criteria that were commonly met (i.e., at or above 75% of studies meeting criteria) included using valid and reliable predictor and outcomes variables (i.e., distress expression and distress regulation behaviors), using continuous predictor variables, accounting for relevant confounding variables, specifying the research questions, specifying the statistical methods, and providing gender distributions. Of note, in order to be included in the meta-analysis, all studies were required to provide a correlation or a statistic that could be converted into a correlation, and thus 100% of studies would have met this criterion.

Items that reduced quality ratings across studies (i.e., at or below 25% of studies meeting criteria) included not blinding outcome assessors to hypotheses, not properly defining the study population, neglecting to report or achieve a participation rate of 50% or more eligible participants, neglecting to report or uniformly implement recruitment criteria across participants, and not reporting exact *p*-values associated with the correlations.

### 3.4. Synthesis of Results: Relations between Distress Expression and Distress Regulation Behaviors

Table 2 summarizes findings from the nine meta-analyses (separate analyses by age and distress regulation behavior). Supplementary Tables S1–S3 provide individual study findings for interested readers.

#### 3.4.1. Meta-Analyses for Disengagement of Attention Regulation Behaviors by Age

##### First Year of Life

**Figure 3:**
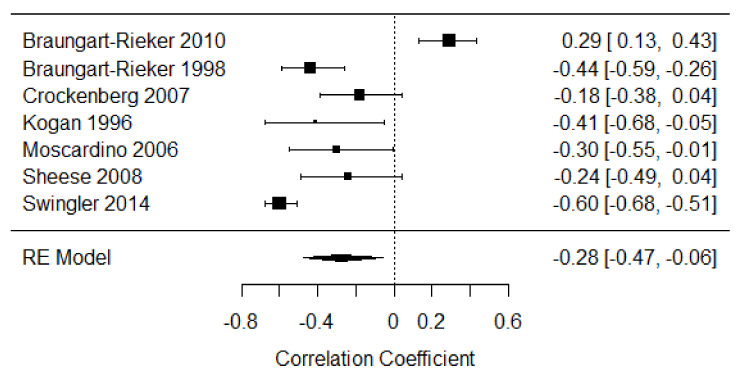
Forest plot of year 1 disengagement of attention. Note: RE = Random-Effects Model.

Seven studies were included in the meta-analysis of disengagement of attention in the first year of life [18,35,36,37,38,39,40] providing a total of 674 participants. The weighted mean effect size was r = −0.28, 95% CI [−0.47, −0.06] indicating a small to moderate negative relationship between distress expression and disengagement of attention behaviors in the first year of life. The heterogeneity of effects among the studies was large (Q = 90.45, *p* < 0.001, I^2^ = 87.18%; see Figure 3).

##### Second Year of Life

**Figure 4:**
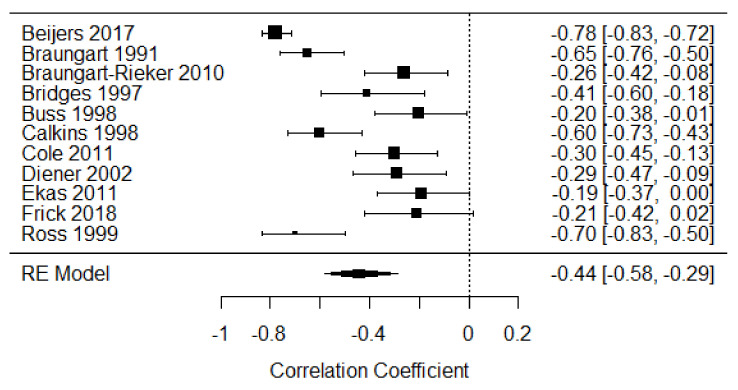
Forest plot of year 2 disengagement of attention. Note: RE = Random-Effects Model.

Eleven studies were included in the meta-analysis of disengagement of attention in the second year of life [14,16,18,25,30,41,42,43,44,45,46], providing 1057 participants. The weighted mean effect size was r = −0.44, 95% CI [−0.58, −0.29], indicating a moderate negative relationship. There was substantial heterogeneity of effects among the studies (Q = 106.48, *p* < 0.001, I^2^ = 88.21%; see Figure 4).

##### Third Year of Life

**Figure 5:**
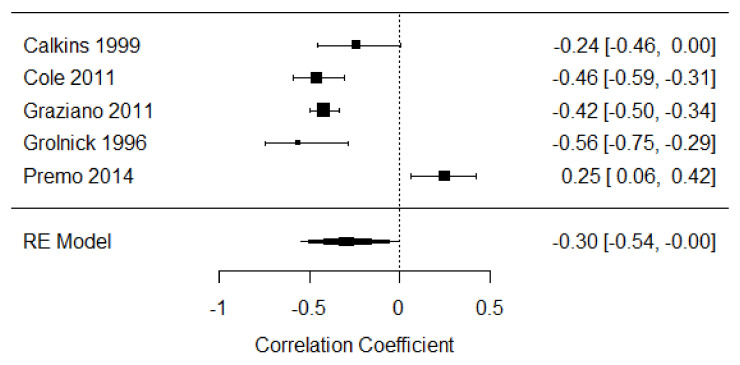
Forest plot of year 3 disengagement of attention. Note: RE = Random-Effects Model.

Five studies were included in the meta-analysis of disengagement of attention in the third year of life [16,17,47,48,49], providing 750 participants. The weighted mean effect size was r = −0.30, 95% CI [−0.54, −0.00], indicating a small to moderate negative relationship between distress expression and disengagement of attention strategies at this age. There was substantial heterogeneity of effects among the studies (Q = 47.72, *p* < 0.001, I^2^ = 92.45%; see Figure 5).

#### 3.4.2. Meta-Analyses for Parent-Focused Strategies by Age

##### First Year of Life

**Figure 6:**
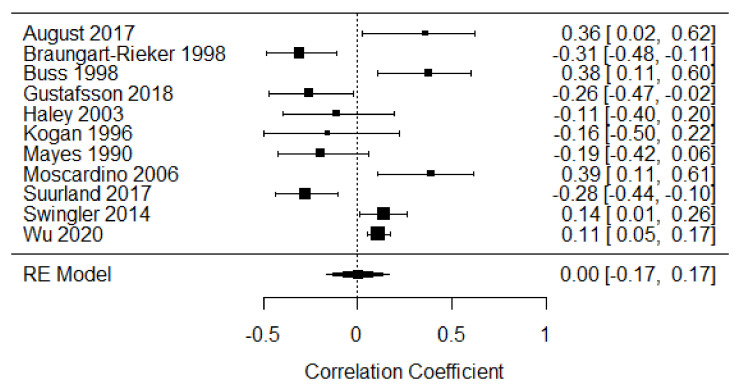
Forest plot of year 1 parent-focused strategies. Note: RE = Random-Effects Model.

Eleven studies were included in the meta-analysis of parent-focused strategies in the first year of life [14,35,37,38,40,50,51,52,53,54,55], providing 1809 participants. The weighted mean effect size was r = 0.00, CI 95% [−0.17, 0.17], indicating a lack of a relationship overall between distress expression and parent-focused strategies. There was large heterogeneity of effects among the studies (Q = 57.52, *p* < 0.001, I^2^ = 88%; see Figure 6).

##### Second Year of Life

**Figure 7:**
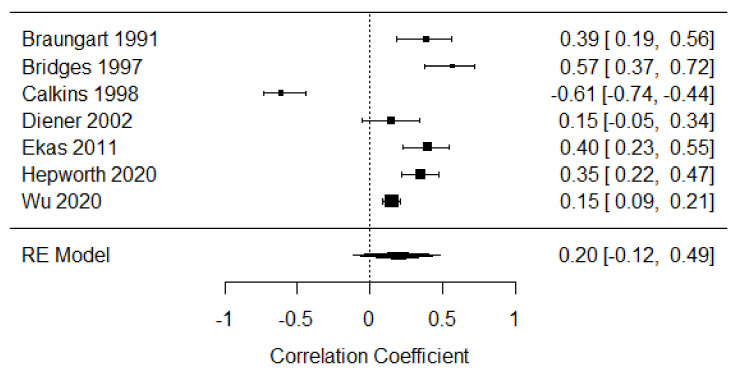
Forest plot of year 2 parent-focused strategies. Note: RE = Random-Effects Model.

Seven studies were included in the meta-analysis of parent-focused strategies in the second year of life [25,42,43,44,45,55,56], providing 1573 participants. The weighted mean effect size was r = 0.20, 95% CI [−0.12, 0.49], indicating a weak positive association between parent-focused behaviors and distress expression. There was substantial heterogeneity across studies (Q = 85.53, *p* < 0.001, I^2^ = 96.50%; see Figure 7).

##### Third Year of Life

**Figure 8:**
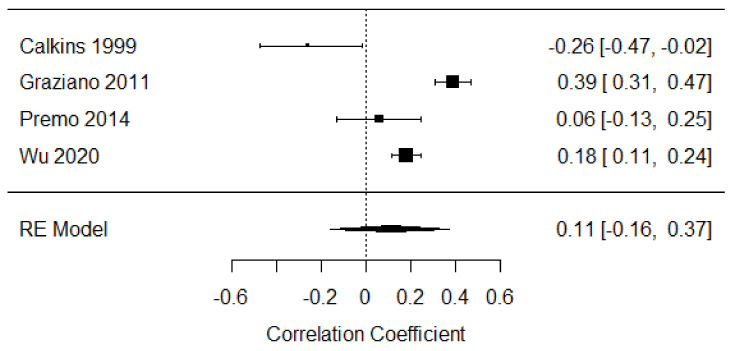
Forest plot of year 3 parent-focused strategies. Note: RE = Random-Effects Model.

Four studies were included in the meta-analysis of parent-focused strategies at this age [47,48,49,55], including data from 1459 participants. The weighted mean effect size was r = 0.11, 95% [−0.16, 0.37], indicating a weak, positive relationship between distress expression and parent-focused strategies. There was large heterogeneity (Q = 33.84, *p* < 0.001, I^2^ = 95.03; see Figure 8).

#### 3.4.3. Meta-Analyses for Self-Soothing Strategies by Age

##### First Year of Life

**Figure 9:**
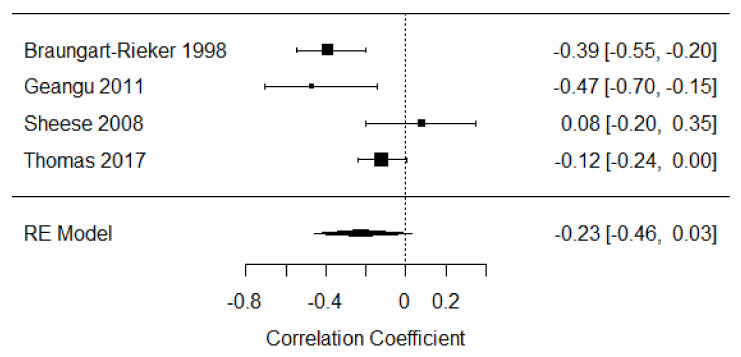
Forest plot of year 1 self-soothing strategies. Note: RE = Random-Effects Model.

Four studies were included in the meta-analysis of self-soothing strategies in the first year of life [23,35,39,57], providing 437 participants. The overall mean effect size was r = −0.23, 95% CI [−0.46, 0.03] indicating a weak negative relationship between distress expression and self-soothing strategies. There was large heterogeneity of effects among studies (Q = 12.10, *p* = 0.007, I^2^ = 81.67%; see Figure 9).

##### Second Year of Life

**Figure 10:**
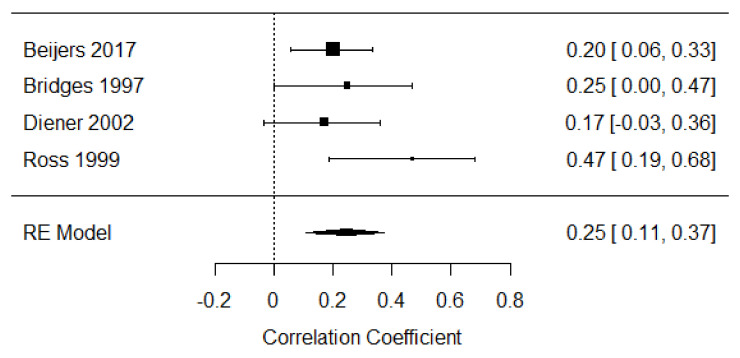
Forest plot of year 2 self-soothing strategies. Note: RE = Random-Effects Model.

Four studies were included in the meta-analysis of self-soothing strategies in the second year of life [25,41,43,46], providing 382 participants. The weighted mean effect size was r = 0.25, 95% CI [0.11, 0.37], indicating a small to moderate positive relationship between distress expression and self-soothing behaviors. Heterogeneity among effects was relatively low (Q =3.38, *p* = 0.34, I^2^ = 41.18%; see Figure 10).

##### Third Year of Life

**Figure 11:**
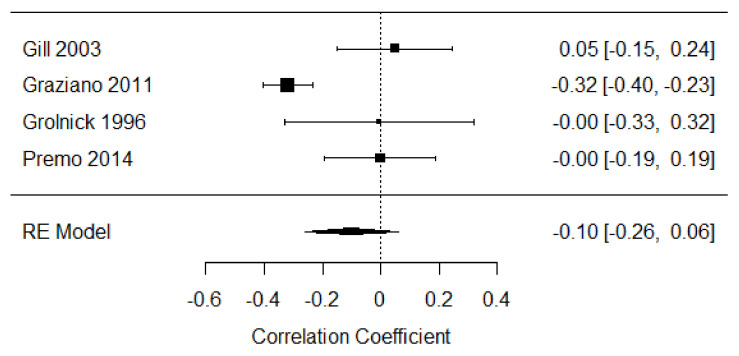
Forest plot of year 3 self-soothing strategies. Note: RE = Random-Effects Model.

Four studies were included in the meta-analysis of self-soothing strategies in the third year of life [17,24,48,49], including data from 664 participants. The weighted mean effect size was r = −0.10, [−0.26, 0.06], indicating a weak, negative relationship between distress expression and self-soothing strategies. There was moderate heterogeneity among studies (Q =18.59, *p* < 0.001, I^2^ = 67.37%; see Figure 11), which was predominantly driven by a single study.

## 4. Discussion

The purpose of this paper was to summarize the literature assessing concurrent relationships between child distress expression and child distress regulation behaviors over the first three years of life. Findings were stratified by age (first year, second year, and third year of life), and cluster of emotion regulation behaviors (disengagement of attention, parent-focused, and self-soothing behaviors). This was done given the evidence of developmental shifts in the first three years between caregiver-directed behaviors and independent self-regulation behaviors, as well as a shift in complexity of independent self-regulation behaviors over time. Disengagement of attention strategies consistently had the strongest relationships across each of the years analyzed. Following is a discussion of these findings in greater detail.

### 4.1. Associations between Distress Expression and Distress Regulation Behaviors

#### 4.1.1. Disengagement of Attention Regulation Behaviors

Over the first three years of life, relationships between distress expression and disengagement of attention were consistently negative, suggesting that greater disengagement of attention was associated with lower levels of behavioral distress. In the first and third year, the magnitude of the negative relationship was small to moderate. In the second year, the magnitude was moderate, suggesting that disengagement of attention is a particularly strong distress regulation behavior during this developmental period. The increase in the strength of these relationships from the first to second year is consistent with Sameroff’s hypothesis [9] that self-led regulatory behaviors become increasingly influential during toddlerhood, and with developmental research that suggests a major shift in attentional capacity throughout the first year, with continued growth across development [4,5,10]. However, the lack of notable increase from the second to third year is less clear. Given that attentional capacity and use of disengagement of attention are expected to increase throughout development, it would be intuitive that the relationship between distress expression and disengagement of attention would be the strongest at three years. While this could be explained by the smaller number of studies available for year three analyses, it is possible that there are more complex cognitive strategies, such as language [19], or other attentional behaviors occurring in the third year that are not captured in the literature summarized here.

#### 4.1.2. Parent-Focused Distress Regulation Behaviors

The relationships between parent-focused distress regulation behaviors and distress expression were consistently small over the first three years of life. Based on the current findings, the strongest relationship is again in the second year of life, with a lack of a relationship in the first year, and a weaker relationship in the third year of life. Findings also suggested that these relationships were positive, indicating that the use of more parent-focused behaviors was related to an increase in distress. This is consistent with early findings of an increase in parent-focused strategies in the second year [10]. The increase in magnitude from the first to second year of life can be understood through Sameroff’s work [9], suggesting an increase in use of more child-directed emotion regulation behaviors from infancy to toddlerhood. The reduction in magnitude from the second to third year is consistent with the shift from more parent-focused strategies to potentially more independently driven strategies as the child develops [4,5,8,9].

The positive direction of these relationship can also be understood as signals from a child to the parent that they need support [6]. Looking across the age groups, in the first year of life, the lack of a relationship may best be understood from an attachment perspective. Parent-focused regulatory behaviors may not be expected to be implemented consistently by children until the attachment relationship between parent and child is more stable, after the first year of life [12,13]. This can help to explain the increase in relationship between parent-focused strategies and distress expression in the second year of life once the attachment relationship and the child’s understanding of how the parent will respond has been solidified. However, it is difficult to fully understand the relationships between parent-focused regulation behaviors and distress across development when the parental response and sensitivity to the displayed distress is left unaccounted. It is possible that it is not the actual signal that would result in a reduction in distress, but rather what the parent does with that signal. Enhancing these findings through analyses that account for the caregiver response would allow for a deeper understanding of the association between distress expression and parent-focused regulation behaviors.

#### 4.1.3. Self-Soothing Distress Regulation Behaviors

In the first and third year of life, there were small negative relationships between self-soothing behaviors and distress expression. Although in the expected negative direction, given that in the first year of life distress regulation is heavily influenced by caregiver behavior [4,5,9], and limited by the child’s developmental stage, it is understandable that the negative association is small. There was a small to moderate positive relationship between self-soothing behaviors and distress expression in the second year of life, which is consistent with the developmental trend of movement away from physical self-soothing to other forms of self-regulation [4,5,10]. The findings of this analysis may be showing that these types of behaviors no longer support distress reduction.

Lastly, the small negative relationship in the third year of life warrants attention as this can be puzzling within the developmental context discussed above. Upon further review of the variability of the data in this analysis, it appears that the majority of findings in this area indicate a lack of a relationship, which would be more consistent with the developmental trends discussed above; however, given the small number of studies in this area, less confidence should be attributed to these effect sizes until more work in the area is completed and synthesized.

To summarize across strategies, in line with developmental theorizing [4,5,8,9], our findings reiterate the need for assessing emotion regulation developmentally across the first few years of life. As children age, we expect their use of emotion regulation strategies to become more sophisticated, and we tend to see a shift from relying on a caregiver to regulate distress, to initiating more independent self-regulation strategies. Our work did not necessarily follow this pattern, as arguably the most complex distress regulation could be the disengagement of attention strategies, and this proved to be the most consistently significant effect size from year one to year three. Additionally, it is possible that language accounts for a lack of findings in year three, which was not measured as a regulation strategy in this review. However, our findings do follow particular trends, notably, a peak in the magnitude of the relationship between emotion regulation behaviors and distress expression in the second year of life, across all three clusters of emotion regulation behaviors. This speaks to the second year being a particularly important time for the use of these behaviors, irrespective of whether the behaviors are related to a reduction or increase in distress.

### 4.2. Heterogeneity across Studies and Methodological Considerations

The above findings need to be understood in the context of substantial heterogeneity among the effect sizes in each analysis. Except for the meta-analysis of self-soothing strategies in the second year of life, there was moderate to large variability across effect sizes included in the various analyses. This finding is important to keep in mind when interpreting the weighted mean effect sizes, because the effect from a given individual study is likely to differ from that mean substantially. Further, an example of how this variability can skew interpretation of findings is seen in the analysis of self-soothing strategies in the third year of life. If one only looked at the mean weighted effect size, it would be concluded that there is a small negative relationship between self-soothing and distress. However, when looking at the spread, a different story suggests that this may be largely affected by one study, with other studies more consistently finding a lack of a relationship.

There was considerable variability in the choice of measures and behaviors included across studies. Distress expression can be measured using several indicators, including crying, fussing, additional facial actions, and body movements. Some studies looked at combinations of these behaviors to indicate distress expression, whereas others examined one in isolation—usually crying or vocal indicators of distress. Regarding the distress regulation behaviors used, how researchers measured different categories of emotion regulation behaviors also varied across studies. Again, some may have only measured one indicator of a particular category, whereas others may have measured numerous behaviors within that category. For example, some studies may have only used visual orientation toward a parent as a parent-focused strategy, whereas other studies may have included visual orientation, gesturing, and vocalizations toward the parent as separate indicators of parent-focused strategies. All of these sources of variability underscore the important topic of how emotion regulation is being studied. Researchers should conduct psychometric analyses on child distress expression and child distress regulation behaviors and adopt a consistent way of measuring this across distress tasks. Without more consistency in measurement and context, a clear synthesis of this literature will likely evade the field.

In terms of the overall quality of this literature, several factors were found to consistently reduce quality across studies. These included lack of reporting on the study population, recruitment consistency across participants, and low (or lack of reports on) participation rates. Perhaps most notably, the most commonly omitted piece of information that impacted quality ratings was the failure to report blinding of assessors to study hypotheses. Given the risk for subjectivity in coding and potential for bias, this is an extremely important methodological consideration. While it is possible that coders were in fact blind to the hypotheses, and the authors simply did not include this in the Methods section, this is necessary information for critically evaluating research findings and speaks to a larger issue—a need for researchers to use a more structured, consistent method of reporting observational and cohort studies in the field. For example, the Strengthening in the Reporting of Observational Studies in Epidemiology (STROBE) Statement [58,59] provides guidelines that may help strengthen the reporting of observational studies in emotion regulation research. Further, more research assessing the validity and reliability of quality and risk of bias measures based on these reporting standards is required.

### 4.3. Limitations and Future Directions

First, all studies included in the meta-analyses included experimental distress tasks implemented either at a research laboratory or in participants’ homes. Experimental procedures are limited to studying distress regulation behaviors in the context of low to moderate distress, given that it is unethical to keep a child in high distress unnecessarily. Naturalistic procedures that invoke distress are likely the only context to observe these extreme levels of distress. A common naturalistic procedure that occurs on a routine basis throughout childhood is vaccination. This context provides a naturalistic stressor that invokes high levels of distress but is a routine part of a child’s development. With rare exception [60], the associations between behaviors such as child disengagement of attention, parent-focused, and self-soothing and distress in this context have yet to be studied in this age range and remain a fruitful area for future research.

It is important to consider these findings within the sociocultural context of the included studies. The research studies included are almost exclusively from the United States, and therefore the findings in the meta-analyses may not be entirely generalizable to other populations. Future research may wish to understand the impact on sociocultural factors on how different emotion regulation behaviors relate to distress.

Another potential limitation is that our inclusion criteria only allowed for studies that examined disengagement of attention strategies (not including use of parent), parent-focused strategies, and self-soothing strategies as distinct emotion regulation behavior clusters. This decision was driven by our goal of comparing the relative importance of these theoretically distinct behavior clusters; however, as a result, any study that observed self-focused strategies that collapsed across attentional and physical self-soothing strategies was excluded (e.g., [15]). Thus, other research groups may have classified emotion regulation behaviors differently. Further, this review and meta-analysis excluded physiological and biological indices of distress. Given the breadth of papers on this topic, we decided to focus solely on observable emotion expression behaviors. Future studies could explore the biological components of emotional regulation to provide a more complete biopsychosocial perspective.

Another important limitation is the focus on concurrent relationships, which does not allow for an understanding of how distress regulation and distress expression behaviors influence one another in a sequential and interactive manner over time, as recommended by Cole and colleagues [1]. To gain a more nuanced understanding of emotion regulation, more work is needed that examines how distress regulation behavior and distress expression are temporally associated. Analyses such as cross-lagged path models continue to be explored, particularly in naturalistic settings that facilitate observing varying levels of distress (e.g., [61]).

## 5. Conclusions

Emotion regulation has been an important topic in the developmental psychology literature for decades. This work is the first meta-analysis to examine the association between child distress regulation behaviors and child distress expression. Using an exhaustive search strategy and high standards of synthesis methodology, this review found that the strongest and most consistent relationships in the literature involved disengagement of attention strategies. Small to moderate relationships were found in year 1, 2, and 3. The strongest relationship was seen in the second year. Parent-focused strategies consistently had positive associations with distress, reflecting their function of signalling distress to the caregiver, and peaked in the second year. The associations for self-soothing behaviors were less consistent but support a reduction in usefulness for reducing distress in the second and third year. Heterogeneity of outcome measures and tasks likely contributed to the weaker and inconsistent findings. Efforts to reduce heterogeneity (e.g., consistency in measurement of behaviors) across studies are needed to create a coherent picture regarding the development of child regulation behaviors and distress. This body of work summarizing the overall concurrent relationships between child distress regulation behaviors and child distress expression has also elucidated an important gap in the literature, specifically, a sparsity of studies assessing these processes in naturalistic high-distress environments that would allow for larger variability in distress. Future research should examine these relationships from a biopsychosocial perspective, within naturalistic distressing situations, to gain a deeper understanding of the development of emotion regulation strategies and their impact on distress.

## Figures and Tables

**Table 1:** Study characteristics.

Study: First Author Last Name (Year)	Country	Sample Size (Year of Life: N)	Distress Task	Task Classification (Fear/ Frustration)	Distress Regulation Behaviors Measured	Overall Quality (% of Items Rated as ‘Yes’ out of Applicable Items)
August (2017)	Canada	Y1: 34	Still face	Fear	PF	67%
Beijers (2017)	Netherlands	Y2: 186	Strange situation procedure	Fear	DoA SS	60%
Braungart (1991)	United States	Y2: 80	Strange situation Procedure— reunion	Fear	DoA PF	67%
Braungart-Rieker (1998)	United States	Y1: 94	Still face	Fear	DoA PF SS	57%
Braungart-Rieker (2010)	United States	Y1: 143 Y2: 119	Gentle arm restraint	Frustration	DoA	67%
Bridges (1997)	United States	Y2: 62	Parent-passive delay with mother (combines the gift and food delay procedures)	Frustration	DoA PF SS	64%
Buss (1998)	United States	Y1: 48 Y2: 103	Y1: Unpredictable mechanical dog Y2: Attractive toy behind barrier	Y1: Fear Y2: Frustration	Y1: PF Y2: DoA	60%
Calkins (1998)	United States	Y2: 73	High chair restraint	Frustration	DoA PF	60%
Calkins (1999)	United States	Y3: 65	Combined 2 frustration tasks: high chair task and barrier (toy in a box) task	Frustration	DoA PF	67%
Cole (2011)	United States	Y2: 120 Y3: 120	Wait task	Frustration	DoA	60%
Crockenberg (2007)	United States	Y1: 80	Novelty to bumble ball and firetruck	Fear	DoA	57%
Diener (2002)	United States	Y2: 94	Competing demands task with mom	Frustration	DoA PF SS	67%
Ekas (2011)	United States	Y2: 106	Parent-ignore- toddler-situation (PITS) with mother, ignore episode—a modified still face *	Frustration	DoA PF	60%
Frick (2018)	Sweden	Y2: 74	Attractive toy placed behind a barrier	Frustration	DoA	43%
Geangu (2011)	Romania	Y1: 32	Emotional resonance task (cry sound of peer)	Fear	SS	54%
Gill (2003)	United States	Y3: 99	Experimenter distress	Fear	SS	57%
Graziano (2011)	United States	Y3: 422	Combined 2 frustration tasks: high chair task and prize in a box task	Frustration	DoA PF SS	57%
Grolnick (1996)	United States	Y3: 37	Separation from parent alone	Fear	DoA SS	67%
Gustafsson (2018)	United States	Y1: 68	Arm restraint	Frustration	PF	64%
Haley (2003)	United States	Y1: 43	Modified still-face protocol: Second still face	Fear	PF	60%
Hepworth (2020)	United States	Y2: 186	Mask task	Fear	PF	88%
Kogan (1996)	United States	Y1: 29	Still face reunion	Fear	DoA PF	53%
Mayes (1990)	United States	Y1: 62	Still face	Fear	PF	53%
Moscardino (2006)	Italy	Y1: 45	Arm restraint	Frustration	DoA PF	73%
Premo (2014)	United States	Y3: 106	Novelty to spider	Fear	DoA PF SS	64%
Ross (1999)	United States	Y2: 40	Attractive toy	Frustration	DoA SS	57%
Sheese (2008)	United States	Y1: 50	Mask procedure	Fear	DoA SS	43%
Suurland (2017)	The Netherlands	Y1: 117	Still face procedure—reunion	Fear	PF	60%
Swingler (2014)	United States	Y1: 233	Arm restraint	Frustration	DoA PF	57%
Thomas (2017)	Canada	Y1: 261	8 frustration trials collapsed	Frustration	SS	75%
Wu (2020)	United States	Y1: 1036 Y2: 972 Y3: 866	Y1: Combined arm restraint task, mask task, and barrier task Y2: Combined mask task and toy removal task Y3: Combined mask task and toy removal task	Frustration	PF	80%

Note. Y1 = first year of life (3 months to 11 months inclusive), Y2 = second year of life (12 months to 23 months inclusive), Y3 = third year of life (24 months to 35 months inclusive), DoA = disengagement of attention, PF = parent-focused strategies, and SS = self-soothing strategies. * All separation tasks were classified as inducing fear. However, Ekas (2011) described their task as inducing frustration, and therefore it was classified accordingly.

**Table 2:** Summary of meta-analysis.

Year of Life	Disengagement of Attention	Parent Focused	Self-Soothing
Year 1	r = −0.28 (k = 7) *	r = 0.00 (k = 11)	r = −0.23 (k = 4)
Year 2	r = −0.44 (k = 11) *	r = 0.20 (k = 7)	r = 0.25 (k = 4) *
Year 3	r = −0.30 (k = 5) *	r = 0.11 (k = 4)	r = −0.10 (k = 4)

Note. Effect estimates (Pearson *r*) and number of studies included in each effect estimate (*k*) are presented. * Indicates the 95% confidence Interval did not cross over 0.

## Data Availability

Publicly available datasets were analyzed in this study. This data can be found here: https://yorkspace.library.yorku.ca/xmlui/handle/10315/38510; accessed on 11 November 2021.

## Supplementary Materials

The following are available online at https://www.mdpi.com/article/10.3390/children9020174/s1, Table S1: Summary of results from studies assessing disengagement of attention separated by age; Table S2: Summary of results from studies assessing parent-focused strategies separated by age; Table S3: Summary of results from studies assessing self-soothing strategies separated by age; File S1. PRISMA checklist; File S2. Systematic search strategies; File S3. Quality assessment measure.
