# Supplementary material for: Child Distress Expression and Regulation Behaviors: A Systematic Review and Meta-Analysis
Source: Children (Basel). 2022 Feb 1;9(2):174. doi: 10.3390/children9020174 (PMC8870305; doi:10.3390/children9020174)
Supplement: Supplementary file 1 [file children-09-00174-s001.zip › children-1482637-supplementary/Supplemental File S2_Systematic Search Strategies.pdf]

Supplemental File S2: Systematic Search Strategies

Search Results

**Database [Platform]** *January 25, 2018*	**Results**
**MEDLINE**<br>Ovid MEDLINE(R) Epub Ahead of Print, In-Process & Other Non-Indexed Citations, Ovid MEDLINE(R) Daily and Ovid MEDLINE(R) 1946 to Present	6674
**Embase Classic+Embase** 1974 to 1947 to 2019 Week 04	8825
**PscyINFO**	3208

MEDLINE

Database(s): **Ovid MEDLINE(R) and Epub Ahead of Print, In-Process & Other Non-Indexed Citations and Daily** 1946 to January 25, 2019
Search Strategy:

#	Searches	Results
1	emotions/ or expressed emotion/ or (emotion or emotions or emotional).tw,kf.	191679
2	stress, psychological/ or (stress or stresses).tw,kf.	751508
3	irritable mood/ or (irritable or irritability).tw,kf.	23335
4	affect/ or (affect or mood or moods or negative reactivity or sad or sadness).tw,kf.	690904
5	anger/ or (tantrum or tantrums or anger*).tw,kf.	17399
6	fear/ or fear*.tw,kf.	83195
7	frustration/ or frustrat*.tw,kf.	17500
8	pain/ or acute pain/ or (pain or pains or distress*).tw,kf.	711933
9	or/1-8	2236871
10	child, preschool/ or exp infant/ or pediatrics/	1527594
11	(infan* or newborn* or "new born*" or perinat* or neonat* or baby or baby* or babies or toddler* or prematur* or pre-term or preterm*).mp.	1563213
12	or/10-11	1987661
13	(regulation or regulatory).tw,kf.	1039331
14	9 and 12 and 13	6674

Embase

Database(s): **Embase Classic+Embase** 1947 to 2019 Week 04
Search Strategy:

#	Searches	Results
1	emotion/ or (emotion or emotions or emotional).tw,kw.	264584
2	mental stress/ or (stress or stresses).tw,kw.	954790
3	irritability/ or (irritable or irritability).tw,kw.	52347
4	affect/ or (affect or mood or moods or negative reactivity or sad or sadness).tw,kw.	885570

Supplemental File S2: Systematic Search Strategies

5	anger/ or (tantrum or tantrums or anger*).tw,kw.	26078
6	fear/ or fear*.tw,kw.	115395
7	frustration/ or frustrat*.tw,kw.	21682
8	pain/ or (pain or pains or distress*).tw,kw.	1096725
9	or/1-8	3062910
10	child, preschool/ or exp infant/ or pediatrics/	1395661
11	(infan* or newborn* or "new born*" or perinat* or neonat* or baby or baby* or babies or toddler* or prematur* or pre-term or preterm*).mp.	1716033
12	or/10-11	1999185
13	(regulation or regulatory).tw,kw.	1290962
14	9 and 12 and 13	8825

PsycINFO

Database(s): **PsycINFO** 1806 to January Week 3 2019
Search Strategy:

#	Searches	Results
1	emotions/ or (emotion or emotions or emotional).tw,id.	293849
2	psychological stress/ or (stress or stresses).tw,id.	213337
3	(irritable or irritability).tw,id.	8761
4	(affect or mood or moods or negative reactivity or sad or sadness).tw,id.	256250
5	anger/ or (tantrum or tantrums or anger*).tw,id.	30121
6	fear/ or fear*.tw,id.	78467
7	frustration/ or frustrat*.tw,id.	17706
8	pain/ or (pain or pains or distress*).tw,id.	152930
9	or/1-8	844282
10	preschool students/ or pediatrics/	33499
11	(infan* or newborn* or "new born*" or perinat* or neonat* or baby or baby* or babies or toddler* or prematur* or pre-term or preterm*).mp.	147940
12	or/10-11	178212
13	(regulation or regulatory).tw,id.	102997
14	9 and 12 and 13	3208
