# Supplementary material for: Child Distress Expression and Regulation Behaviors: A Systematic Review and Meta-Analysis
Source: Children (Basel). 2022 Feb 1;9(2):174. doi: 10.3390/children9020174 (PMC8870305; doi:10.3390/children9020174)
Supplement: Supplementary file 1 [file children-09-00174-s001.zip › children-1482637-supplementary/Supplemental File S3_Quality.pdf]

Supplemental File S3: Quality Assessment Measure

INSTRUCTIONS

The task of assessing the quality of individual studies is part of assessing the strength of the body of evidence under review. In order to evaluate the overall strength of evidence put forth in our systematic review, we are using a modification of the checklists designed by the National Heart, Blood, and Lungs Institute (2014; https://www.nhlbi.nih.gov/health-pro/guidelines/in-develop/cardiovascular-risk-reduction/tools/cohort), Downs and Black (1998) and Crombie (1996).

The attached excel sheet will include the 31 studies with 16 questions to be answered as either Yes, No or Not Applicable (NA). The questions will be answered by thoroughly reading each article. A final proportion score (total endorsed items out of total applicable items) will be made based on the instructions on the last page.

Disagreements between reviewers will be discussed with a third reviewer (RPR). From the discussion, a final score will be determined for each paper.

To expedite the process of discussion, for each article reviewed, please electronically or manually label where you have identified the answer to the particular question posed below (i.e., by writing Q1 or Q2, etc., in the margin beside where supporting information regarding each QA question was identified).

QUALITY ASSESSMENT CHECKLIST

1. **Was the research question or objective in this paper clearly stated?**
   - Usually in the last paragraphs of the Introduction, you will notice that the author will describe the study and what their research questions/aims/objectives are for the current study.
   - For example, "The objective of this study was to…"
   - Possible responses: yes/no

2. **Was the study population clearly specified and defined?**
   - All three criteria are important to consider:
     - Did the authors specify whether it was a normative / clinical / abused / SES at risk sample?
     - Did the authors specify what country or city participants were recruited from?
     - Did the authors specify when the participants were recruited (i.e., between what time points)
   - For example:
     - Participants included a normative sample of mother-child dyads recruited between January and December of 1990, from the USA.
   - This information is usually found within the "Participants" section.
   - Possible responses: yes/no

3. **Was the participation rate of eligible persons at least 50%?**
   - Does the study describe the number of eligible people?
   - If fewer than 50% of eligible persons participated in the study, then there is concern that the study population does not adequately represent the target population. This increases the risk of bias.
   - You are comparing the number of people who were approached with the number of people

who agreed to participate in the study. If this information is not provided at all, the answer should be "no".

- Note: If the required information is not included in the current article, but another paper is cited that has more detailed information about the study sample, please go to that paper to see if the criteria is met.
- Even if the paper is analyzing one wave of a longitudinal study, it's still important to know that the initial recruited sample was representative of the population it's supposed to be representing. If this information is not mentioned, or if another paper with more details is not cited, the answer is "no".
- Possible responses: yes/no

4.

- **4A. Were all the subjects selected or recruited from the same or similar populations (including the same time period)?**
    - If two groups of participants are being compared (e.g., children of depressed mothers vs. children of non-depressed mothers), were both participant groups recruited from the same population?
    - If all participants in the study were recruited using the same means (for example, a community mail-out), and if you have no reason to believe that participants were recruited from different samples, then the answer to this question would be "yes".
    - If the authors did not specify the time period during which participants were recruited, the answer to this question would be "no".
    - Please note: this needs to be discussing the population that was used for our specific analyses.
    - Possible responses: yes/no
- **4B. Were inclusion and exclusion criteria for being in the study pre-specified and applied uniformly to all participants?**
    - There are two criteria that must be met in order to answer "yes" to this question:
        1. Were the inclusion/exclusion criteria determined ahead of time and used to screen participants during recruitment, or were they determined *after* recruitment and used to exclude participants who had been recruited already?
        2. Were the same inclusion/exclusion criteria used for all participants in the study?
    - Note: this question ***is not*** asking whether or not inclusion/exclusion criteria were used. It is ensuring that, if inclusion/exclusion criteria were used, they were used planned in advance and used consistently across subjects.
    - Possible responses: yes/no/NA
    - Note: If no inclusion/exclusion criteria are described, the answer to this question should be "NA".

5. Was a sample size justification, power description, variance accounted for or effect estimates provided?

- Did the authors present their reasons for selecting or recruiting the number of people included or analyzed? Do they note or discuss the statistical power, variance accounted for or effect estimates of the study?
- A paragraph in the methods section of the article may explain the sample size needed to detect a hypothesized difference in outcomes. You may also find a discussion of power in the discussion section (e.g., the study had 85 percent power to detect a 20 percent increase in the rate of an outcome of interest, with a 2-sided alpha of 0.05).

- Sometimes estimates of variance accounted for and/or estimates of effect size are given, instead of sample size calculations. In any of these cases, the answer would be "yes."
- This information may be found in the “Participants” or “Results” section.
- Possible answers: yes/no

6. **For <u>predictor variables</u> (e.g., child self-soothing) that can vary in amount or level, did the study examine different levels of the predictor/examine as a continuous variable as related to the outcome?**
   - Is it possible to investigate a dose-response relationship for the child level factors? And if yes, was a dose-response relationship investigated? For example, this would involve comparing the relationship between emotion regulation behaviours and negative affect as continuous variables. If the authors used a dichotomous variable they would receive a “No”.
   - Possible answers: yes/no/NA

7. **Were the <u>predictor variables</u> clearly defined, valid, reliable, and implemented consistently across all study participants?**
   - For studies where the strategies are clearly described, the question should be answered “yes”. For studies which refer to previous research supporting this claim, or that demonstrate the outcome measures are reliable within their own sample (i.e., reporting interrater reliability), the question should be answered “yes.”
   - Our criteria for inter-rater reliability include:
     - Minimum 75% agreement between coders <u>*or*</u>
     - Kappa equal to or greater than 0.5.
     - Minimum .80 ICC or correlation
   - As long as the study’s coder training criteria or actual agreement/ Kappa values meet **<u>at least one</u>** of our criteria, the study receives credit for this item.
   - If inter-rater reliability was not reported on, or if it did not meet our criteria, the answer to this question is “no”.
   - Possible answers: yes/no

8. **Was the <u>predictor variable</u> assessed more than once over time?**
   - Multiple measurements with the same result increase our confidence that the predictor variable was correctly classified.
   - Possible answers: yes/no

9. **Were measures of the <u>outcome variable</u> clearly defined, valid, reliable, and implemented consistently across all study participants?**
   - Same criteria as #7.
   - Possible answers: yes/no

10. **Were the outcome assessors (e.g. negative affect/distress coders) blinded to the study hypotheses?**
    - Is it likely that the person collecting data or coding would know or would be able to figure out the study hypotheses?
    - Look for a line, usually in the Methods section, that states that coders/editors were blind to study hypotheses. If this isn’t stated, answer should be “no”.
    - Possible answers: yes/no
11. **Was loss to follow-up after baseline 20% or less?**
    - Note: This study is specific to longitudinal studies. If the study is not longitudinal,

it automatically receives "NA" for this item.

- Typically, an acceptable overall follow-up rate is considered to be 80% or more.
- The information you are looking for is whether the authors have included a participation rate that represents how many participants were recruited vs. how many participated in all study procedures.
- If there isn't a rate calculated, but the authors give the number of non-participants, participants lost to follow up, or participants with missing data who were excluded from analyses, you can calculate it using a simple calculation.
- For example: 64 mother-child dyads participated at Time 1 and 55 participated at Time 2. 55/64 = 85% therefore, they would get a "yes" since only 15% (or 100-85) was lost to follow-up.
- Possible answers: yes/no/NA

12. Were key potential confounding variables measured? Was the relationship adjusted statistically for their impact on the relationship between exposure(s) and outcome(s)?

- For the purposes of this research, key potential confounding variables may include:
    - Age, sex, SES, maternal characteristics
    - **Note:** as long as ***one*** of the above mentioned variables is included then they get a point
- Was the relationship between these variables and outcomes investigated? If significantly associated with outcomes, were the variables statistically adjusted for?
- Note: we want to know whether authors *considered* the influence of these variables on outcomes. If they investigated the relationship between gender and outcomes, found that there was no relationship, and thus did not control for gender, they still receive credit here (and the answer here should be "yes").
- Possible answers: yes/no

13. Is the distribution of the overall study population by gender described?

- Have the authors included how many male and female children are in the sample.
- The information may be found in the demographics/participant characteristics table; however, some studies will include it in the "Participants" or "Results" section.
- Possible answers: yes/no

14. Are the statistical methods described?

- The information you need is whether they have described what type(s) of analyses (e.g., correlations, regression) were conducted.
- This information can be found in the "Statistical Analysis" or "Results" section.
- Possible answers: yes/no

15. Have actual probability values been reported (e.g., 0.035 rather than < 0.05) for the main outcomes except where the probability value is less than 0.001?*

- This information can be found in the "Results" section or results tables.
- We are only focusing on the statistics for the analyses that we are interested in for the purposes of this review paper (i.e., those to child emotion regulation and negative affect/distress). For these analyses, we expected to see p values reported regardless of whether or not the analysis was significant.
- If probability values are all less than 0.001, then mark as a "yes"
- Possible answers: yes/no
