# Supplementary material for: Child Distress Expression and Regulation Behaviors: A Systematic Review and Meta-Analysis
Source: Children (Basel). 2022 Feb 1;9(2):174. doi: 10.3390/children9020174 (PMC8870305; doi:10.3390/children9020174)
Supplement: Supplementary file 1 [file children-09-00174-s001.zip › children-1482637-supplementary/Supplemental Table S1_Disengagement of Attention by Age.pdf]

Supplemental Table 1. *Summary of Results from Studies Assessing Disengagement of Attention Separated by Age.*

	Year 1		Year 2		Year 3	
Study	Analysis	Result	Analysis	Result	Analysis	Result
Moscardino (2006)	Correlation	r = -0.30				
Bridges (1997)			Correlation	r = -0.41		
Swingler (2014)	Correlation	r = -0.60				
Beijers (2017)			Correlation	r = -0.78		
Premo (2014)					Correlation	r = 0.25
Ekas (2011)			Correlation	r = -0.19		
Braungart (1991)			Correlation	r = -0.65		
Diener (2002)			Correlation	r = -0.29		
Sheese (2008)	Correlation	r = -0.24				
Cole (2011)			Correlation	r = -0.30	Correlation	r = -0.46
Frick (2018)			Correlation	r = -0.21		
Buss (1998)			Correlation	r = -0.20		
Braungart-Rieker (2010)	Correlation	r = 0.29	Correlation	r = -0.26		

Braungart-Rieker (1998)	Correlation	r = -0.44				
Calkins (1998)			Correlation	r = -0.60		
Grolnick (1996)					Correlation	r = -0.56
Graziano (2011)					Correlation	r = -0.42
Ross (1999)			Correlation	r = -0.70		
Calkins (1999)					Correlation	r = -0.24
Crockenberg (2007)	Correlation	r = -0.18				
Kogan (1996)	Correlation	r = -0.41				
