# Supplementary material for: Child Distress Expression and Regulation Behaviors: A Systematic Review and Meta-Analysis
Source: Children (Basel). 2022 Feb 1;9(2):174. doi: 10.3390/children9020174 (PMC8870305; doi:10.3390/children9020174)
Supplement: Supplementary file 1 [file children-09-00174-s001.zip › children-1482637-supplementary/Supplemental Table S2_Parent-Focused by Age.pdf]

Supplemental Table 2. *Summary of Results from Studies Assessing Parent-Focused Strategies Separated by Age.*

	Year 1		Year 2		Year 3	
Study	Analysis	Result	Analysis	Result	Analysis	Result
Moscardino (2006)	Correlation	r = 0.39				
Bridges (1997)			Correlation	r = 0.57		
Swingler (2014)	Correlation	r = 0.14				
Haley (2003)	Correlation	r = -0.11				
Premo (2014)					Correlation	r = 0.06
Ekas (2011)			Correlation	r = 0.40		
Braungart (1991)			Correlation	r = 0.39		
Diener (2002)			Correlation	r = 0.15		
Suurland (2017)	Correlation	r = -0.28				
Gustafsson (2018)	Correlation	r = -0.26				
August (2017)	Correlation	r = 0.36				
Braungart-Rieker (1998)	Correlation	r = -0.31				
Calkins (1998)			Correlation	r = -0.61		

Graziano (2011)					Correlation	r = 0.39
Buss (1998)	Correlation	r = 0.38				
Mayes (1990)	Correlation	r = -0.19				
Calkins (1999)					Correlation	r = -0.26
Wu (2020)	Correlation	r = 0.11	Correlation	r = 0.15	Correlation	r = 0.18
Kogan (1996)	Correlation	r = -0.16				
Hepworth (2020)			Correlation	r = 0.35		
