# Supplementary material for: Child Distress Expression and Regulation Behaviors: A Systematic Review and Meta-Analysis
Source: Children (Basel). 2022 Feb 1;9(2):174. doi: 10.3390/children9020174 (PMC8870305; doi:10.3390/children9020174)
Supplement: Supplementary file 1 [file children-09-00174-s001.zip › children-1482637-supplementary/Supplemental Table S3_Self-Soothing by Age.pdf]

Supplemental Table 3. *Summary of Results from Studies Assessing Self-Soothing Strategies Separated by Age.*

	Year 1		Year 2		Year 3	
Study	Analysis	Result	Analysis	Result	Analysis	Result
Bridges (1997)			Correlation	r = 0.25		
Beijers (2017)			Correlation	r = 0.20		
Premo (2014)					Correlation	r = 0.00
Diener (2002)			Correlation	r = 0.17		
Geangu (2011)	T Test (Converted)	r = -0.47				
Sheese (2008)	Correlation	r = 0.08				
Thomas (2017)	Correlation	r = -0.12				
Gill (2003)					Correlation	r = 0.05
Braungart-Rieker (1998)	Correlation	r = -0.39				
Grolnick (1996)					Correlation	r = 0.00
Graziano (2011)					Correlation	r = -0.32
Ross (1999)			Correlation	r = 0.47		
